# Continuous diagnostic models for volume deficit in patients with acute diarrhea

**DOI:** 10.1186/s41182-021-00361-9

**Published:** 2021-09-06

**Authors:** J. Austin Lee, Kexin Qu, Monique Gainey, Samika S. Kanekar, Meagan A. Barry, Sabiha Nasrin, Nur H. Alam, Christopher H. Schmid, Adam C. Levine

**Affiliations:** 1 Department of Emergency Medicine, Warren Alpert Medical School, Brown University, 55 Claverick Street, Providence, RI 02903 USA; 2 Department of Biostatistics, Brown University School of Public Health, Providence, RI USA; 3 Rhode Island Hospital, Providence, RI USA; 4 Alpert Medical School, Brown University, Providence, RI USA; 5 International Centre for Diarrhoeal Disease Research, Bangladesh (icddr,b), Dhaka, Bangladesh

**Keywords:** Acute diarrhea, Dehydration, Rehydration, Fluid deficit, Regression model

## Abstract

**Background:**

Episodes of acute diarrhea lead to dehydration, and existing care algorithms base treatment around categorical estimates for fluid resuscitation. This study aims to develop models for the percentage dehydration (fluid deficit) in individuals with acute diarrhea, to better target treatment and avoid the potential sequelae of over or under resuscitation.

**Methods:**

This study utilizes data from two prospective cohort studies of patients with acute diarrhea in Dhaka, Bangladesh. Data were collected on patient arrival, including weight, clinical signs and symptoms, and demographic information. Consecutive weights were obtained to determine the true volume deficit of each patient. Data were entered into two distinct forward stepwise regression logistic models (DHAKA for under 5 years and NIRUDAK for 5 years and over).

**Results:**

A total of 782 patients were included in the final analysis of the DHAKA data set, and 2139 were included in the final analysis of the NIRUDAK data set. The best model for the DHAKA data achieved an *R*^2^ of 0.27 and a root mean square error (RMSE) of 3.7 (compared to *R*^2^ of 0.06 and RMSE of 5.5 with the World Health Organization child care algorithm) and selected 6 predictors. The best performance model for the NIRUDAK data achieved an *R*^2^ of 0.28 and a RMSE of 2.6 (compared to *R*^2^ of 0.08 and RMSE of 4.3 with the World Health Organization adolescent/adult care algorithm) and selected 7 predictors with 2 interactions.

**Conclusions:**

These are the first mathematical models for patients with acute diarrhea that allow for the calculation of a patient’s percentage dehydration (fluid deficit) and subsequent targeted treatment with fluid resuscitation. These findings are an improvement on existing World Health Organization care algorithms.

**Supplementary Information:**

The online version contains supplementary material available at 10.1186/s41182-021-00361-9.

## Background

Acute diarrhea is a major cause of morbidity and mortality and annually leads to an estimated 1.65 million deaths worldwide; it is the fifth highest cause of lost disability adjusted life-years across all ages [1–3]. Although diarrhea is a global problem, the burden of disease is unequally distributed, with a higher burden in low- and middle-income countries (LMICs) that often have fewer healthcare resources [1]. Acute diarrhea is generally defined as stool in greater frequency and less formed than usual lasting for less than 14 days; some definitions require at least three or more bowel movements above usual in less than 24 h [4]. Dehydration is a leading symptom of diarrhea and affects both children and adults [3, 5, 6]. Early diagnosis of dehydration can be challenging, but early and accurate case detection can ameliorate high rates of morbidity and mortality [3, 5, 7].

The management of acute diarrhea is focused on several factors, including an assessment of the severity of the dehydration, a calculation of ongoing fluid losses, and serial monitoring of the patient’s clinical status [8]. Correction of fluid and electrolyte imbalances should drive the modality and duration of treatment [9]. Existing World Health Organization (WHO) guidance splits care of acute diarrhea into under-five (Integrated Management of Childhood Illness, IMCI) and at-or-above 5 years of age (Integrated Management of Adolescent and Adult Illness, IMAI), and recommends treating patients with acute diarrhea with as much oral fluid as they are willing to take until the diarrhea stops [10, 11]. If necessary, intravenous (IV) fluid rehydration is given based solely on a patient’s weight (30 ml/kg as a bolus, followed by 70 ml/kg over several hours) [10, 11]. The risk of underhydration or overhydration and associated sequelae are of particular concern during protocolized treatment, particularly with patients at the extremes of age and patients who are malnourished or have other comorbidities [12–14].

The key to treating dehydration from acute diarrhea is assessing the amount of dehydration and subsequently replenishing a patient’s fluid deficit. Knowledge of the percentage dehydration is important since calculated percentage of dehydration can be used to calculate total fluid deficit:

$${\text{Fluid deficit }}\left( {{\text{liters}}} \right) = {\text{percent dehydration }} \times {\text{ patient weight }}({\text{kilograms}}).$$

This fluid deficit calculation allows providers to quickly and accurately determine the amount of oral and/or IV rehydration a patient will require for appropriate treatment.

At present, there are no multivariable models that accurately predict a patient’s percentage of dehydration or fluid deficit; this study aims to fill this gap. The calculation of the percentage volume deficit from the acute diarrhea can then be used to support health care professionals in the triage, accurate treatment, and ongoing management of these patients particularly in settings with limited resources.

## Methods

### Study design

Data were collected as part of two separate prospective cohort studies among patients with acute diarrhea. Dehydration: Assessing Kids Accurately (DHAKA) studied patients under 5 years of age; Novel, Innovative Research for Understanding Dehydration in Adults and Kids (NIRUDAK) studied patients 5 years of age and older. Both studies have developed clinical diagnostic models for assessing dehydration severity from acute diarrhea using an ordinal outcome (none, some, or severe dehydration). The ordinal logistic regression models for the DHAKA study have undergone both derivation with internal validation and external validation, while the NIRUDAK study has undergone diagnostic model derivation with internal validation, and has planned external validation in 2022 [15–17]. Neither study has reported a predictive model for the amount of fluid deficit measured on a continuous scale.

Ethical approval for the studies was obtained from the International Centre for Diarrhoeal Research, Bangladesh (icddr,b) and the Rhode Island Hospital Institutional Review Boards.

### Study setting and population

Participants were recruited from a single site at the icddr,b Dhaka Hospital rehydration unit 24 h per day, 7 days per week. Data were collected at this site from February to June 2014 for the DHAKA study and March 2019 to March 2020 for the NIRUDAK study. High patient volumes (up to 200 patients per day with acute diarrhea) precluded consecutively screening/enrolling every patient. Marbles were randomly drawn from a blind pouch each time a patient presented to the hospital; if a colored marble was drawn, the patient was screened for enrollment, and if a white marble was drawn, the patient was not screened or enrolled. If a screened patient met eligibility criteria and consent was obtained, the patient was enrolled.

For the DHAKA study, all children under 5 years of age presenting with acute diarrhea were eligible for enrollment; for the NIRUDAK study, patients at least 5 years of age were eligible for inclusion in the study if they presented with symptoms of acute diarrhea. In both studies, patients were excluded if they had been enrolled previously in the study, had fewer than 3 loose stools in 24 h, or had an initial diagnosis other than gastroenteritis as determined by the triage physician. For the DHAKA study, patients were also excluded if they had diarrhea lasting longer than 14 days; in the NIRUDAK study patients were also excluded if they had diarrhea lasting longer than 7 days. For eligible patients, research staff provided the patient and/or their parent/guardian with information about the goals, risks, and benefits of the study and obtained verbal/written consent in the local language, Bangla.

### Study size

The models developed in this paper are secondary analyses of the DHAKA and NIRUDAK datasets; sample sizes for each dataset were calculated, a priori, for use as clinical diagnostic models [15–17].

### Staff training and oversight

For both the DHAKA and NIRUDAK studies, local general practice nurses with several years of clinical experience were hired outside of the icddr,b clinical nursing pool to collect data. Prior to the start of the study, research staff received at least 5 days of didactic and hands-on training in all study procedures, including randomization and the assessment of clinical signs of dehydration under the guidance of the principal investigator, research physicians, and program coordinator.

### Study procedures

After obtaining informed consent, patient’s baseline weights were obtained. For the DHAKA study, children were undressed and weighed using an electronic Seca 383 digital baby/floor scale, while for the NIRUDAK study patients were not undressed and an electronic Seca 952 chair or Seca 984 bed scale was used. All three digital scales measure patient weights to the nearest tenth of a kilogram. Additionally, if a patient received intravenous fluid before a baseline weight was obtained, study staff recorded the amount of fluid received prior to weight measurement to determine the patient’s weight before any fluids were given.

For both studies, patients were independently assessed by two research nurses from a predetermined list of symptoms/signs of dehydration, with each nurse blinded to the other’s clinical assessment. Signs/symptoms of dehydration were chosen a priori based on a review of the literature and consultation with expert clinicians at icddr,b (Additional file 1: Appendix S1 and Appendix S3). Social and demographic information was obtained from either the patient or parent/guardian. For both studies, research procedures were not allowed to delay emergent care, such as placing an intravenous line or giving fluids. After the initial weight and clinical assessment, all patient treatment and clinical care was given according to standard icddr,b protocols. For the DHAKA study, patients were weighed every eight hours to determine a post-hydration stable weight. In the NIRUDAK study, patients were weighed every four hours and similarly measured until a post-hydration stable weight was obtained.

### Dehydration methods

Percent weight change with rehydration was used as the criterion standard for percent dehydration in our study, as described in existing literature [18–21]. As patients with dehydration from acute diarrhea underwent treatment and were rehydrated, their weight increased until they became euvolemic and they began to diurese excess fluid, at which point their body weight stabilized. This stable weight was calculated by averaging the two highest consecutive weight measurements (that differed by less than 2%) [22]. Patients who did not achieve a stable weight prior to being clinically appropriate for discharge were called daily until their diarrhea resolved, then asked to return for a final post-illness weight measurement. In both studies, the percent dehydration was calculated as follows:

$${\text{Percent dehydration}}\, = \,{1}00 \times \left[ {\left( {{\text{post}} - {\text{illness weight}} - {\text{admission weight}}} \right)/{\text{post}} - {\text{illness weight}}} \right].$$

### Model derivation

Models were developed and reported in accordance with the EQUATOR (Enhancing the QUAlity and Transparency Of health Research) Network’s Transparent Reporting of a multivariable prediction model for Individual Prognosis Or Diagnosis (TRIPOD) checklist [23]. All statistical analyses were performed using R Version 3.6.3 [24]. Six different models were developed from 17 candidate predictors for DHAKA and 8 predictors for NIRUDAK (as described previously) [17].

Categorical predictors were modeled using a set of indicator variables relative to a chosen normal reference level. The levels of several categorical predictors with few observations in DHAKA were combined with neighboring levels: dry mucous membranes (very dry was grouped with dry/sticky), respirations (very deep was combined with deep), and heart rate (very fast was grouped with fast).

Continuous variables were modeled both on a linear scale and as restricted cubic splines using knots at the 10th, 50th, and 90th quantiles. In both sets of predictors, we also analyzed some of the continuous variables in a categorical format by grouping their values. These continuous variables were converted to categorical scales to reflect their uneven distributions (clustering around round numbers), and the thresholds were chosen based on both the observed data distribution and the clinical relevance. In DHAKA, we categorized number of diarrheal episodes into 3 levels (< 11, 11–20, > 20), and duration of diarrhea in two formats, one with three levels (< 49, 49–96, > 96) and one with five levels (< 25, 25–48, 49–72, 73–96, > 96). In NIRUDAK, we grouped number of vomiting episodes into 4 levels (none, 1–4, 5–9, > 10), number of diarrheal episodes into 3 levels (3–9,10–19, > 19); and duration of diarrhea into 3 levels (0–12,13–23, > 23 h).

We fit six different models for each dataset and used cross-validation to choose the best form. The six models used: (1) categorical predictors only (including continuous predictors in categorical form); (2) categorical predictors with all two-way interactions; (3) categorical and continuous (linear form) predictors; (4) categorical and continuous (linear form) predictors with all two-way interactions; (5) categorical and continuous predictors (spline form); (6) categorical and continuous predictors (spline form) with all two-way interactions).

A forward stepwise regression algorithm was used to select the variables included in each of the six types of models the best model via tenfold cross-validation using mean squared error to choose the number of forward steps to take [17]. The six models were then compared on various performance measures to choose the best one.

### Model performance

Models were examined for adherence to standard linear model assumptions (linearity, normality and homoscedasticity) and were assessed for their accuracy (using *R*^2^ and RMSE) as well as their reliability for predicting percent dehydration. Reliability was assessed by comparing the model predictions from each nurse’s assessment using the Intraclass Correlation Coefficient (ICC). In the DHAKA study, both derivation and validation data were available, so models were validated externally by the RMSE of the model from the training data applied to the external validation dataset. Validation of NIRUDAK models was assessed through bootstrapping with 1000 iterations and over-optimism was corrected in estimating the RMSE [25].

## Results

### Participants and study population characteristics DHAKA

**Fig. 1:**
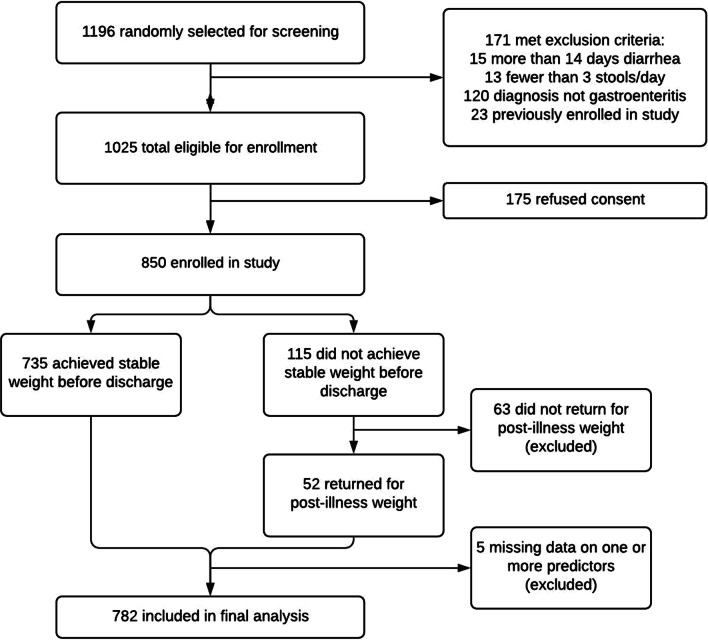
DHAKA enrollment

From February to June 2014 a total of 1196 patients under 5 years presenting to icddr,b with diarrhea were randomly selected for screening, of whom 1025 were eligible and 850 were enrolled. Among those enrolled, 68 patients were excluded for either missing data on outcome (*n* = 63) or input predictors (*n* = 5) leaving 782 patients included in the final analysis (Fig. 1). Median age was 15 months (25th and 75th percentiles 9 and 28.8 months) and 339 patients (43.4%) were female (Table 1). Among enrollees, 219 patients received some IV fluids before admission weight was obtained (median 15 ml, interquartile range 10–20 ml).

**Table 1:** Population characteristics^a^

	DHAKA study (*N* = 782)	NIRUDAK study (*N* = 2139)
Age^b^. median (25–75th percentile)	15 (9–29 months)	35 (18–60 years)
Sex, No. (%)		
Female	339 (43)	1063 (50)
Male	443 (57)	1095 (50)
Home location, No. (%)		
Urban	490 (63)	1628 (76)
Rural/suburban	291 (37)	511 (24)
Nutritional status (MUAC)^c^, No. (%)		
Severe wasting	35 (5)	31 (1)
Moderate wasting	121 (15)	164 (8)
No wasting	626 (80)	1944 (91)
Diarrhea duration and frequency, median (25–75th percentiles)		
Days of diarrhea Prior to arrival	2 (1–4)	0 (0–1)
Loose stools Prior 24 h	15 (10–20)	15 (10–20)
Dehydration category, No. (%)		
Severe dehydration	84 (11)	277 (13)
Some dehydration	344 (44)	1431 (67)
No dehydration	354 (45)	431 (20)

^a^Categorical variables were summarized as number (percent), continuous variables summarized as median (25–75th percentile

^b^For DHAKA Study, age is presented in months; for the NIRUDAK Study, age is presented in years

^c^Nutritional status was calculated using the mid-upper arm circumference (MUAC) as outlined in the literature (26–28). For the DHAKA study, the proportion of children with undernutrition was determined using a MUAC < 115 mm for severe wasting and 115–125 mm for moderate wasting and > 125 for normal. For the NIRUDAK Study, patients between the ages of 5–9 years old were categorized as severe wasting if MUAC was < 135 mm, moderate wasting if MUAC was 135–145 mm and normal if MUAC measurement was > 145 mm. Patients between the ages of 10–14 years old were categorized as severe wasting if MUAC measurement was < 160 mm, moderate wasting if MUAC measurement was 160–185 mm and normal if MUAC was > 185 mm. For patients 15 years of age and older, severe wasting was defined as a MUAC measurement < 185 mm; moderate wasting was categorized as a MUAC 185–210 mm; normal was defined as a MUAC measurement > 210 mm

### Participants and study population characteristics NIRUDAK

**Fig. 2:**
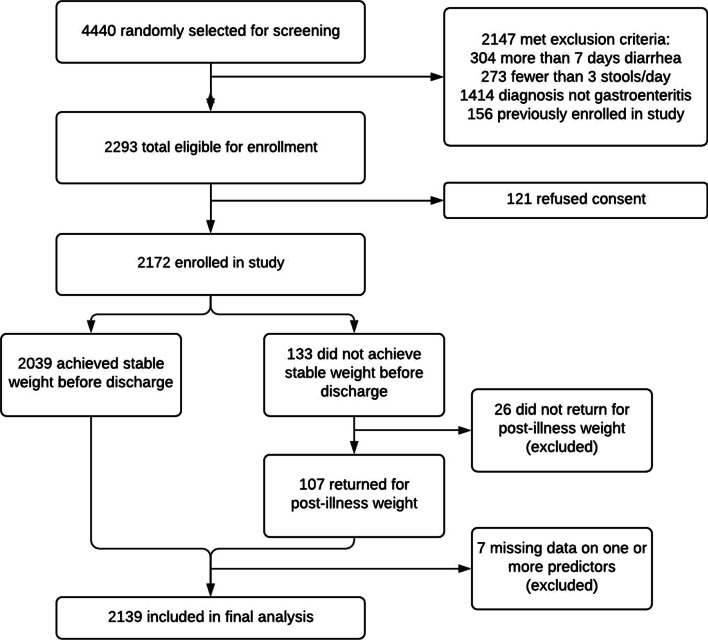
NIRUDAK enrollment

From March 2019 to March 2020, a total of 4,440 patients over 5 years presenting to icddr,b with diarrhea were randomly selected for screening, of whom 2293 were eligible and 2172 were enrolled. Of these, 33 were excluded from analysis for missing data, including final post-illness weight (*n* = 26) or one or more predictors (*n* = 7) leaving 2139 included in the final analytic dataset (Fig. 2). This dataset included 630 children (age 5–19), 748 adults (age 20–59), and 761 elderly patients (age ≥ 60) (Fig. 2). Median age was 35 years (25th and 75th percentiles of 18 and 60 years) and 1063 patients (49.7%) were female (Table 1). Among enrollees, four patients received any IV fluids before admission weight was obtained (one patient each received 10 ml, 100 ml, 0.5 l, and 1 l).

### Model performance

On the training data, the best model for the DHAKA data achieved an *R*^2^ of 0.27 and a RMSE of 3.7 and selected six predictors (general appearance, skin pinch, tears, radial pulse, age in months, and number of diarrheal episodes in the past 24 h) (Table 3, Additional file 1: Appendix S1 and S2). The best model for the NIRUDAK data achieved an *R*^2^ of 0.28 and a RMSE of 2.6 and contained seven predictors [sunken eyes, skin pinch, vomiting episodes in the past 24 h, sex, age in years, systolic blood pressure when lying flat, and mid-upper arm circumference (MUAC)] with 2 interactions of age and vomiting episode, and skin pinch and MUAC (Table 4, Additional file 1: Appendix S3 and S4). The reliability, as measured by the ICC was 0.88 (95% CI 0.85–0.90) for the DHAKA model and 0.93 (95% CI 0.92–0.94) for the NIRUDAK model.

The *R*^2^ and RMSE of the DHAKA and NIRUDAK forward stepwise regression models were contrasted with the values attained from instead using the existing standard of care WHO IMCI and IMAI algorithms (Table 2).

**Table 2:** R^2^ and RMSE of forward stepwise regression models vs WHO algorithm

	*R*^2^		RMSE	
	Model	WHO	Model	WHO
DHAKA	0.27	0.06	3.7	5.5
NIRUDAK	0.28	0.08	2.6	4.3

**Fig. 3:**
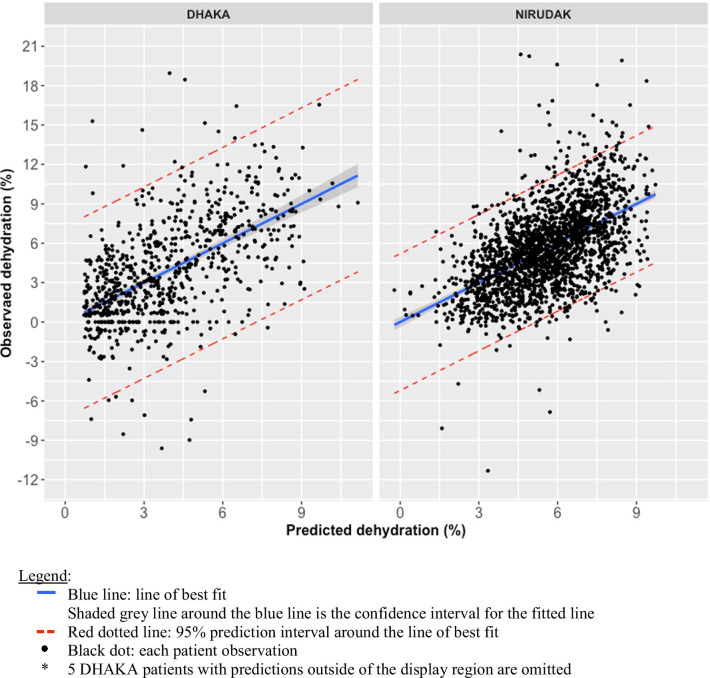
Observed vs. predicted plot, DHAKA* and NIRUDAK models

Figure 3 plots the observed versus predicted percent dehydration from the final models constructed on the training data. The blue line of best fit showed that the model was well-calibrated on average (i.e., the average of the observed dehydration equals the predicted dehydration at each level of predicted dehydration). The red 95% predicted lines, which included 95% of the individual observed values at each prediction level, showed that for a given predicted dehydration value, we expect 95% of the observed values to lie within about 5% of the predicted values. The figure also showed the observed versus predicted percent dehydration from the final models constructed on the training data.

### Model validation

RMSE on the validation of the DHAKA data was 3.73. The average optimism corrected RMSE of the NIRUDAK model across 1000 bootstrap iterations was 2.72 (95% CI 2.59–2.84).

## Discussion

### Interpretation of results

Both the DHAKA and NIRUDAK models for the assessment of percentage of dehydration from acute diarrhea are the first multivariate models to allow for the calculation of a patient’s fluid deficit. Previous tools have used categorical assessment and treatments which are generally a reasonable approach to care. Yet, every patient with acute diarrhea will have a different level of dehydration and require a unique amount of rehydration. Both models were found to be an improvement on the existing WHO standard of care algorithms.

Clinical application of the models developed here would entail that one would first select the appropriate model based on the patient’s age (less than 5 years, or 5 years and up) and after the requisite predictor inputs are then measured/obtained, the resultant output value represents the percentage of dehydration in that given patient. The patient’s weight (kg) is then multiplied by the percent dehydration to obtain the fluid deficit (in liters) required for fluid rehydration, along with monitoring for ongoing fluid losses to maintain adequate treatment.

An accurate assessment of percentage dehydration and fluid deficit is valuable in targeting resuscitation, particularly when contrasted with the current standard of care WHO IMCI and IMAI algorithms. These algorithms recommend fluid resuscitation volumes based solely on a patient’s weight (broadly, 75 ml per kilogram of oral fluid or 100 ml per kg of IV fluid). These therapeutic approaches do not hone the treatment to the extent of an individual’s fluid deficit [10, 11]. Furthermore, categorical tools and algorithms lend themselves to the risk of underhydration and overhydration. Patients at the extremes of age (very young and elderly) as well as the critically ill and malnourished are at particular risk of the sequalae from either underhydration or overhydration. While weight-based treatment can be useful and at times adequate, our models allow for more accurate treatment and allow providers to better avoid the ill effects of incorrect rehydration.

While the clinical data collection methods used were performed by trained study nurses at a single site, the modeling input variables required to assess a patient’s fluid deficit should be able to be obtained by healthcare providers with basic levels of education and clinical knowledge. It is hoped that these findings will be generalizable to other LMICs, where the burden of morbidity and mortality of acute diarrhea is particularly high.

### Model performance

One strength of this study is that both prediction models have limited missing data, particularly the NIRUDAK study. As noted in Table 2, both the DHAKA and NIRUDAK models are an improvement over the existing standard of care WHO algorithms; the models both have a higher *R*^2^ and lower RMSE than the WHO approach to care. The *R*^2^ is a measure of the percentage of total variation in the dependent variable that is accounted for by the independent variable [29]. Yet, in human population studies, there is no set threshold or methodology for interpreting the *R*^2^, and models with *R*^2^ values of 0.25 and lower are common [29].

Plots of the observed versus predicted values for each model showed that the line of best fit is quite accurate in predicting a patient’s percent dehydration against the observed dehydration, on average (as seen in the narrow grey shaded area around the lines of best fit in Fig. 3). While individual patient level prediction does have a range across a prediction interval, we believe that the ability to predict an individual’s volume deficit for diarrhea is, in fact, a significant improvement on the categorical existing standard of care that does not account for differences between individuals. There are few outlying observations beyond the prediction intervals as noted by the RMSE (standard error for prediction) for each plot. Both models are slightly less accurate at the upper extremes of predicting more severe dehydration. This finding of a regression towards the mean is expected with a forward stepwise regression model.

The included predictors (Tables 3, 4) for each model had limited overlap which reinforces the necessity of delineating between patients above and below 5 years of age, as is current practice with WHO management protocols. Only skin pinch and age were incorporated into both models. Skin pinch or skin turgor is not a perfect corollary for dehydration, but does have diagnostic utility as a clinical sign of dehydration in both children and adults [30–32]. Further, the contrast in the distinct categorical predictors between the two models is notable (general appearance, tears, and radial pulse for DHAKA, and sunken eyes, sex, and episodes of vomiting for NIRUDAK). Continuous variables also had significant contrasting predictors (number of diarrhea episodes past 24 h for DHAKA, versus MUAC and systolic BP while supine in NIRUDAK).

**Table 3:** Predictors and regression coefficients in the DHAKA model

Categorical variables, No. (%)		Regression coefficient (95% CI)	*P* value
General appearance			
Normal (reference level)	394 (50)	1	0.002
Restless/irritable	176 (23)	0.38 (− 0.20, 1.23)	0.157
Lethargic/unconscious	212 (27)	1.14 (0.62, 2.20)	0.001
Skin pinch			
Rapid (reference level)	384 (49)	1	< 0.001
Slow	325 (42)	1.29 (1.12, 2.37)	< 0.001
Very slow	73 (9)	6.30 (1.74, 4.01)	< 0.001
Tears			
Present (reference level)	374 (48)	1	0.01
Decreased	328 (42)	0.47 (− 0.14, 1.08)	0.132
Weak/absent	80 (10)	1.59 (0.57, 2.62)	0.002
Radial pulse			
Strong (reference level)	524 (67)	1	0.05
Decreased	115 (15)	0.91 (0.06, 1.76)	0.036
Absent	143 (18)	0.92 (0.03, 1.80)	0.042
Continuous variables, mean, (SD)			
Age (months)	20.0 (14.5)	− 0.12 (− 0.18, − 0.05)	< 0.001
Number of diarrhea Episodes, past 24 h	16.8 (8.2)	− 0.02 (− 0.11, 0.07)	0.671

**Table 4:** Predictors and regression coefficients in the NIRUDAK model

Categorical variables, No. (%)		Regression coefficient (95% CI)	*P* value
Eye level			
Normal (reference level)	546 (26)	1	< 0.001
Sunken	1593 (74)	0.90 (0.62, 1.19)	
Skin pinch			
Rapid (reference level)	825 (39)	1	0.591
Slow	1053 (49)	1.14 (− 1.69, 3.97)	0.428
Very slow	261 (12)	− 0.93 (− 6.17, 4.30)	0.727
Vomiting episodes in 24 h			
None (reference level)	208 (10)	1	< 0.001
1–5	729 (34)	2.55 (0.76, 4.33)	0.005
6–10	770 (36)	4.09 (2.34, 5.84)	< 0.001
> 10	432 (20)	3.94 (2.11, 5.78)	< 0.001
Sex			
Male	336 (44)	1	< 0.001
Female	435 (56)	0.69 (0.46, 0.92)	
Continuous variables, mean, (SD)			
Age (years)	38.3 (22.1)	0.13 (0.06, 0.19)	< 0.001
Systolic BP (supine)	94.2 (20.6)	− 0.01 (− 0.02, 0.01)	0.398
MUAC	236.4 (36.7)	− 0.02 (− 0.03, − 0.01)	0.005

In both the NIRUDAK and DHAKA studies, only about 1–2% of all patients enrolled had an observed negative percent dehydration (representing weight loss between arrival and final post-illness weight), and in nearly all cases these negative values were very close to 0 (almost all from 0 to − 2%). These likely represent minor weighing errors or rounding errors in our weight assessments for patients with very minimal to no dehydration despite having acute diarrhea. Our ICC findings indicate good-to-excellent reliability in both cohorts, and study methods were designed to minimize potential measurement errors. However, patients were only weighed to the nearest 0.1 kg and a stable weight was considered to be two consecutive weights within 2% of each other such that there may be a small variance in a given patient’s observed dehydration calculation.

### Limitations

Our study has several limitations. First, data were collected at a single site, at icddr,b’s Dhaka Hospital. While this hospital has a large catchment area and diverse patient population, it is not necessarily representative of all other LMIC nor high-income contexts. Furthermore, our data were collected as part of two distinct study protocols with different enrollment dates. While study protocols were not identical in both studies, each model was developed based on only one data set or the other. Study data were collected by trained nurses, who had at least some prior nursing experience, but were also early in their careers. Model inputs were based on clinical and demographic data that can be easily obtained at the patient’s bedside with limited training. While findings and protocols may not be ideally generalizable to other clinical assessors such as lesser trained community health workers, the study was designed to maximize generalizability to the extent possible. Furthermore, the DHAKA model had both derivation and validation data sets available while the NIRUDAK study data only included a derivation data set and will have forthcoming data for subsequent validation in the near future. Additional future work will need to evaluate the clinical and economic value of targeted rehydration strategies based on fluid deficit as opposed to preexisting categorical assessments of dehydration.

## Conclusions

This paper puts forth a secondary analysis of data from two distinct but related prospective cohorts of patients with acute diarrhea from Dhaka, Bangladesh. Based on forward stepwise regression models, the measurement of several demographic and clinical data points allows for the calculation of a percentage dehydration and associated fluid deficit in patients with acute diarrhea. These are the first such models to allow for the calculation of a patient's fluid deficit in a way that can subsequently be utilized to target rehydration therapy more accurately, particularly when compared to categorical WHO algorithms.

The clinical application of these models is to take a patient with acute diarrhea and accurately predict the patient’s volume deficit to then guide clinicians in how much intravenous or oral fluid should be given to the patient in the clinic or hospital setting. It is hoped that there are potential cost savings and reduced morbidity from unnecessary overtreatment for patients with lower fluid deficit, while more robust resuscitation can be targeted at patients with increased dehydration to ameliorate morbidity and mortality. Our expectation is that the clinical care of children, adolescents, and adults with acute diarrhea will be enhanced by the accurate calculation of fluid deficit and resultant targeted clinical resuscitation.

## Supplementary Information

**Additional file 1. S1 Appendix**. Pre-defined protocols for measurement of all clinical variables in the DHAKA Model.** S2 Appendix**. DHAKA Formula.** S3 Appendix**. Pre-defined protocols for measurement of all clinical variables in the NIRUDAK Model.** S4 Appendix**. NIRUDAK Formula.

## Data Availability

The data supporting the conclusions of this article are available in the Open Science Framework repository, https://osf.io/pncms/.

## Abbreviations

icddr,b: International Centre for Diarrhoeal Research, Bangladesh
RMSE: Root mean square error
LMICs: Low- and middle-income countries
WHO: World Health Organization
IMCI: Integrated management of childhood illness
IMAI: Integrated management of adolescent and adult illness
IV: Intravenous
DHAKA: Dehydration: assessing kids accurately
NIRUDAK: Novel, innovative research for understanding dehydration in adults and kids
EQUATOR: Enhancing the quality and transparency of health research
TRIPOD: Transparent reporting of multivariable prediction model for individual prognosis or diagnosis
ICC: Intraclass correlation coefficient
MUAC: Mid-upper arm circumference

## References

[1] Troeger C, Blacker BF, Khalil IA, Rao PC, Cao S, Zimsen SR (2018). Estimates of the global, regional, and national morbidity, mortality, and aetiologies of diarrhoea in 195 countries: a systematic analysis for the Global Burden of Disease Study 2016. Lancet Infect Dis.

[2] Vos T, Lim SS, Abbafati C, Abbas KM, Abbasi M, Abbasifard M (2020). Global burden of 369 diseases and injuries in 204 countries and territories, 1990–2019: a systematic analysis for the Global Burden of Disease Study 2019. The Lancet.

[3] Vega RM, Avva U. Pediatric dehydration. In: StatPearls [Internet]. Treasure Island (FL): StatPearls Publishing; 2020 [cited 2021 Jan 22]. http://www.ncbi.nlm.nih.gov/books/NBK436022/.

[4] Riddle MS, DuPont HL, Connor BA (2016). ACG clinical guideline: diagnosis, treatment, and prevention of acute diarrheal infections in adults. Am J Gastroenterol.

[5] Miller HJ (2015). Dehydration in the older adult. J Gerontol Nurs.

[6] Silver AJ (1990). Aging and risks for dehydration. Cleve Clin J Med.

[7] Weinberg AD, Minaker KL (1995). Dehydration. Evaluation and management in older adults. Council on Scientific Affairs, American Medical Association. JAMA.

[8] Ofei SY, Fuchs GJ (2019). Principles and practice of oral rehydration. Curr Gastroenterol Rep.

[9] Reber E, Gomes F, Dähn IA, Vasiloglou MF, Stanga Z (2019). Management of dehydration in patients suffering swallowing difficulties. J Clin Med.

[10] World Health Organization, UNICEF. Handbook IMCI: Integrated Management of Childhood Illness. World Health Organization; 2005. 210 p.

[11] World Health Organization. Integrated Management of Adolescent and Adult Illness (IMAI) district clinician manual: hospital care for adolescents and adults: guidelines for the management of common illnesses with limited resources. Thatcher RW, Röhr-Rouendaal P, L’IV Com Sàrl, editors. Geneva, Switzerland: World Health Organization; 2011. 2 p.

[12] World Health Organization, Department of Child and Adolescent Health and Development. The treatment of diarrhoea: a manual for physicians and other senior health workers. Geneva: Dept. of Child and Adolescent Health and Development, World Health Organization; 2005.

[13] Srivastava VK, Arya H, Uppal SS, Rath B, Laisram N (1985). Comparison of oral and intravenous rehydration among hospitalized children with acute diarrhoea. J Diarrhoeal Dis Res.

[14] Hew-Butler T, Smith-Hale V, Pollard-McGrandy A, VanSumeren M (2019). Of mice and men-the physiology, psychology, and pathology of overhydration. Nutrients.

[15] Levine AC, Glavis-Bloom J, Modi P, Nasrin S, Rege S, Chu C (2015). Empirically derived dehydration scoring and decision tree models for children with diarrhea: assessment and internal validation in a prospective cohort study in Dhaka, Bangladesh. Global Health: Sci Practice.

[16] Levine AC, Glavis-Bloom J, Modi P, Nasrin S, Atika B, Rege S (2016). External validation of the DHAKA score and comparison with the current IMCI algorithm for the assessment of dehydration in children with diarrhoea: a prospective cohort study. Lancet Glob Health.

[17] Levine AC, Barry MA, Gainey M, Nasrin S, Qu K, Schmid CH (2021). Derivation of the first clinical diagnostic models for dehydration severity in patients over five years with acute diarrhea. PLOS Negl Trop Dis.

[18] Hooper L, Abdelhamid A, Attreed NJ, Campbell WW, Channell AM, Chassagne P (2015). Clinical symptoms, signs and tests for identification of impending and current water-loss dehydration in older people. Cochrane Database Syst Rev.

[19] Cheuvront SN, Ely BR, Kenefick RW, Sawka MN (2010). Biological variation and diagnostic accuracy of dehydration assessment markers. Am J Clin Nutr.

[20] Kavouras SA (2002). Assessing hydration status. Curr Opin Clin Nutr Metab Care.

[21] Shirreffs SM (2003). Markers of hydration status. Eur J Clin Nutr.

[22] Gorelick MH, Shaw KN, Murphy KO (1997). Validity and reliability of clinical signs in the diagnosis of dehydration in children. Pediatrics.

[23] Collins GS, Reitsma JB, Altman DG, Moons KGM (2015). Transparent reporting of a multivariable prediction model for individual prognosis or diagnosis (TRIPOD): the TRIPOD statement. Ann Intern Med.

[24] R Core Team (2020). R: a language and environment for statistical computing [Internet]. Vienna, Austria: R Foundation for Statistical Computing; https://www.R-project.org/.

[25] Harrell FE, Lee KL, Mark DB (1996). Multivariable prognostic models: issues in developing models, evaluating assumptions and adequacy, and measuring and reducing errors. Stat Med.

[26] World Health Organization (WHO), United Nations Children’s Fund (UNICEF). Child growth standards and the identification of severe acute malnutrition in infants and children [Internet]. Geneva: World Health Organization; 2009 [cited 2021 Mar 9] p. 11. http://www.who.int/nutrition/publications/severemalnutrition/9789241598163/en/. 24809116

[27] Cashin K, Oot L. Guide to Anthropometry: a practical tool for program planners, managers, and implementers. Washington (DC): Food Nutr Tech Assist III Proj (FANTA)/ FHI 360; 2018 May p. 231.

[28] James WP, Mascie-Taylor GC, Norgan NG, Bistrian BR, Shetty PS, Ferro-Luzzi A (1994). The value of arm circumference measurements in assessing chronic energy deficiency in Third World adults. Eur J Clin Nutr.

[29] Hamilton DF, Ghert M, Simpson AHRW (2015). Interpreting regression models in clinical outcome studies. Bone Joint Res.

[30] de Vries Feyens C, de Jager CPC. Decreased Skin Turgor [Internet]. 10.1056/NEJMicm1005144. Massachusetts Medical Society; 2011 [cited 2021 Apr 8]. https://www.nejm.org/doi/10.1056/NEJMicm1005144.

[31] Popov T (2005). Review: capillary refill time, abnormal skin turgor, and abnormal respiratory pattern are useful signs for detecting dehydration in children. Evid Based Nurs.

[32] Fayomi O, Maconochie I, Body R (2007). Is skin turgor reliable as a means of assessing hydration status in children?. Emerg Med J.

