# Correction: Continuous diagnostic models for volume deficit in patients with acute diarrhea

**DOI:** 10.1186/s41182-022-00451-2

**Published:** 2022-09-01

**Authors:** J. Austin Lee, Kexin Qu, Monique Gainey, Samika S. Kanekar, Meagan A. Barry, Sabiha Nasrin, Nur H. Alam, Christopher H. Schmid, Adam C. Levine

**Affiliations:** 1 Department of Emergency Medicine, Warren Alpert Medical School, Brown University, 55 Claverick Street, Providence, RI 02903 USA; 2 Department of Biostatistics, Brown University School of Public Health, Providence, RI USA; 3 Rhode Island Hospital, Providence, RI USA; 4 Alpert Medical School, Brown University, Providence, RI USA; 5 International Centre for Diarrhoeal Disease Research, Bangladesh (icddr,b), Dhaka, Bangladesh

## Correction to: Trop Med Health (2021) 49:70 https://doi.org/10.1186/s41182-021-00361-9

Following publication of the original article [1], the authors reported some errors in Tables 3 and 4 of the manuscript. The reference levels within Tables 3 and 4 should be 0 instead of 1. Cutoff points for vomiting episodes in Table 4 should be 1–4, 5–9, > 9. The reference level of sex should be female in Table 4, and the coefficient for male is 0.69.

The correct Tables 3 and 4 have been provided in this Correction.

**Table 3:** Predictors and regression coefficients in the DHAKA model

Categorical variables, No. (%)		Regression coefficient (95% CI)	*P* value
General appearance			0.002
Normal (reference level)	394 (50)	0	
Restless/irritable	176 (23)	0.38 (− 0.20, 1.23)	0.157
Lethargic/unconscious	212 (27)	1.14 (0.62, 2.20)	0.001
Skin pinch			< 0.001
Rapid (reference level)	384 (49)	0	
Slow	325 (42)	1.29 (1.12, 2.37)	< 0.001
Very slow	73 (9)	6.30 (1.74, 4.01)	< 0.001
Tears			0.01
Present (reference level)	374 (48)	0	
Decreased	328 (42)	0.47 (− 0.14, 1.08)	0.132
Weak/absent	80 (10)	1.59 (0.57, 2.62)	0.002
Radial pulse			0.05
Strong (reference level)	524 (67)	0	
Decreased	115 (15)	0.91 (0.06, 1.76)	0.036
Absent	143 (18)	0.92 (0.03, 1.80)	0.042

Continuous variables, Mean, (SD)			
Age (months)	20.0 (14.5)	− 0.12 (− 0.18, − 0.05)	< 0.001
Number of diarrhea episodes, past 24 h	16.8 (8.2)	− 0.02 (− 0.11, 0.07)	0.671

**Table 4:** Predictors and regression coefficients in the NIRUDAK model

Categorical variables, No. (%)		Regression coefficient (95% CI)	*P* value
Eye level			
Normal (reference level)	546 (26)	0	
Sunken	1593 (74)	0.90 (0.62, 1.19)	< 0.001
Skin pinch			0.591
Rapid (reference level)	825 (39)	0	
Slow	1053 (49)	1.14 (− 1.69, 3.97)	0.428
Very slow	261 (12)	− 0.93 (− 6.17, 4.30)	0.727
Vomiting episodes in 24 h			< 0.001
None (reference level)	208 (10)	0	
1–4	729 (34)	2.55 (0.76, 4.33)	0.005
5–9	770 (36)	4.09 (2.34, 5.84)	< 0.001
> 9	432 (20)	3.94 (2.11, 5.78)	< 0.001
Sex			
Female	435 (56)	0	
Male	336 (44)	0.69 (0.46, 0.92)	< 0.001

Continuous variables, Mean, (SD)			
Age (years)	38.3 (22.1)	0.13 (0.06, 0.19)	< 0.001
Systolic BP (supine)	94.2 (20.6)	− 0.01 (− 0.02, 0.01)	0.398
MUAC	236.4 (36.7)	− 0.02 (− 0.03, − 0.01)	0.005

The original article [1] has been corrected.
