# Supplementary material for: Continuous diagnostic models for volume deficit in patients with acute diarrhea
Source: Trop Med Health. 2021 Sep 6;49:70. doi: 10.1186/s41182-021-00361-9 (PMC8422628; doi:10.1186/s41182-021-00361-9)
Supplement: Supplementary file 1 — Additional file 1. S1 Appendix. Pre-defined protocols for measurement of all clinical variables in the DHAKA Model. S2 Appendix. DHAKA Formula. S3 Appendix. Pre-defined protocols for measurement of all clinical variables in the NIRUDAK Model. S4 Appendix. NIRUDAK Formula. [file 41182_2021_361_MOESM1_ESM.docx]

**S1 Appendix. Pre-defined protocols for measurement of all clinical variables in the DHAKA Model.**

General Appearance

General appearance or mental status was assessed throughout the examination both while the child was being held in the arms of their parent or guardian and while undergoing study procedures such as weight and MUAC assessments. The child was classified as “Normal” if they were awake and alert, acting appropriately for their age (i.e., smiling, looking around, crying at appropriate times but easily consolable by their parent). The child was classified as “Restless/Irritable” if the child was awake but crying or moving almost constantly and difficult for their parent or guardian to console. The child was classified as “Lethargic/Unconscious” if they were either slow to respond or did not respond at all to the nurse’s voice or touch. The child’s eyes may be closed, or they have been open, but the child does not follow anyone with their eyes.

Skin Pinch

A skin pinch test was performed on the child by grasping a fold of skin on the side of their abdomen between the thumb and index finger (at least a few centimeters to the left or right of the umbilicus) and rapidly releasing the skin while counting how many seconds it took for the skin to flatten again. “Normal” was defined by the skin flattening immediately (in the blink of an eye). “Slow” was defined by the skin flattening in about one second. “Very Slow” was defined by the skin flattening in two or more seconds.

Tears

Tears was evaluated by watching the child cry and looking to see whether they produced any tears. If the child did not cry at some point during the baseline procedures, the nurse asked the parent/guardian if their child had tears present during crying at any point in the past few hours. The patient’s tears were classified as “Normal” if the child had tears clearly run down their face, “Decreased” if tears were not visible on the face but could be seen in the eyes by pulling back the lower eyelid and “Absent” if no tears or moisture were visible when pulling back the lower eyelid.

Radial Pulse

Radial pulse was evaluated by placing two fingers just proximal to the child’s wrist crease on the radial side or “them side” of the forearm, pressing just enough to feel the pulse but not too hard. The radial pulse was classified as “Strong” if the radial pulse could be felt easily with a “bounce” during each beat. The radial pulse was classified as “Decreased” if the radial pulse could be felt but is diminished and has no bounce. The radial pulse was classified as “Weak” if the radial pulse could be felt only intermittently or not at all.

Age

The patient’s age was reported by the child’s guardian.

Diarrheal Episodes in 24 hours

Diarrheal episodes in 24 hours were assessed by asking the child’s guardian how many discrete episodes of diarrhea the patient had in the past 24 hours.

**S2 Appendix. DHAKA Formula**

Percent Dehydration = 2.81 + 0.51 (if general appearance = restless) + 1.41 (if general appearance = lethargic) + 1.75 (if skin pinch = slow) + 2.88 (if skin pinch = very slow) + 0.47 (if tears = decreased)

+ 1.59 (if tears = absent) + 0.91 (if radial pulse = decreased) + 0.92 (if radial pulse = weak/absent)

– 0.12 (age, in months) + 0.3 ($x_{\text{agemonths' }}$) – 0.02 (episodes of diarrhea in 24 hours) + 0.12 ($x_{\text{episodes' }}$)

Note:

$$\begin{matrix} x_{\text{agemonths' }} & =\left[ \frac{x_{\text{agemonths }}-7}{10.7} \right]_{+}^{3}+0.30\cdot\left[ \frac{x_{\text{agemonths }}-42}{10.7} \right]_{+}^{3}-1.30\cdot\left[ \frac{x_{\text{agemonths }}-15}{10.7} \right]_{+}^{3} \\ x_{\text{episodes' }} & =\left[ \frac{x_{\text{episodes }}-8}{7.85} \right]_{+}^{3}+0.47\cdot\left[ \frac{x_{\text{episodes }}-30}{7.85} \right]_{+}^{3}-1.47\cdot\left[ \frac{x_{\text{episodes }}-15}{7.85} \right]_{+}^{3} \\ \text{ where }[x]_{+} & =\left\{ \begin{matrix} x & \text{ if }x>0 \\ 0 & \text{ otherwise } \end{matrix} \right. \end{matrix}$$

**S3 Appendix. Pre-defined protocols for measurement of all clinical variables in the NIRUDAK Model.**

Eye Level

The patient’s eye level was evaluated by viewing the patient’s face from the side of the stretcher at the level of the patient and identifying whether the patient’s eyelid was below their orbital rim with their eyes closed. If so, their eye level was classified as “Sunken”, otherwise it was classified as “Normal.” If it was unclear based on visualization, nurses were instructed to place the lateral aspect of one finger across the patient’s orbital rim, with their finger touching both the superior and inferior portions of their orbital rim while the patient’s eyes were closed. The eye level was classified as “Normal” when the nurse could feel the eyelid touching their finger and “Sunken” when the eyelid was below the level of the orbital rim and not touching their finger.

Skin Pinch

A skin pinch test was performed on the patient by grasping a fold of skin on the side of their abdomen between the thumb and index finger and rapidly releasing the skin while counting how many seconds it took for the skin to flatten again. “Rapid” was defined by the skin flattening immediately (in the blink of an eye). “Slow” was defined by the skin flattening in about one second. “Very Slow” was defined by the skin flattening in two or more seconds.

Vomiting Episodes in 24 hours

Vomiting episodes in 24 hours were assessed by asking the patient or their family member how many discrete episodes of vomiting the patient had in the past 24 hours.

Sex

The patient’s sex was reported by the patient and/or guardian.

Age

The patient’s age was reported by the patient and/or guardian.

Systolic Blood Pressure

The patient’s blood pressure was obtained while the patient was lying flat using an automated blood pressure cuff. If the patient was receiving IV fluids, the arm opposite of the IV line was used so as to not interfere with treatment. If the automatic blood pressure cuff was not able to obtain a measurement on the first try, a manual cuff was used instead. For children, a manual, child-sized blood pressure cuff was used to measure blood pressure.

MUAC

The mid-upper arm circumference (MUAC) was assessed by bending the patient’s left elbow to 90 degrees while their left arm was hanging loosely at their side (not stretched out) and measuring the midpoint between the tip of the shoulder and the tip of the elbow. A standard MUAC tape was wrapped around the arm at the measured midpoint, and the observed number was recorded in millimeters.

**S4 Appendix. NIRUDAK Formula**

Percent Dehydration = 4.64 + 0.91 (if eyes = sunken) + 1.14 (if skin pinch = slow)

– 0.93 (if skin pinch = very slow) + 2.55 (vomiting episodes in 24 hours, if 1-5)

+ 4.09 (vomiting episodes in 24 hours, if 6-10) + 3.94 (vomiting episodes in 24 hours, if >10) + 0.69 (if sex = male) + 0.13 (age in years) – 0.17 ($x_{\text{age }},$) – 0.01 (systolic blood pressure when supine) – 0.01 ($x_{SysBP'}$) – 0.02 (MUAC) – 0.08 (age in years * vomiting episodes in 24 hours, if 1-5) + 0.09 ($x_{\text{age}},$ * vomiting episodes in 24 hours, if 1-5) – 0.13 (age in years * vomiting episodes in 24 hours, if 6-10) + 0.14 ($x_{\text{age}},$ * vomiting episodes in 24 hours, if 6-10) – 0.11 (age * vomiting episodes in 24 hours, if >10) + 0.14 ($x_{\text{age}},$ * vomiting episodes in 24 hours, if >10) + 0.02 (MUAC * 1, if skin pinch = very slow) – 0.05 ( $x_{MUAC'}$ * 1, if skin pinch = very slow)

Note:

$$\begin{matrix} x_{\text{age }}, & =\left[ \frac{x_{\text{age }}-12}{14.11} \right]_{+}^{3}+0.93\cdot\left[ \frac{x_{\text{age }}-65}{14.11} \right]_{+}^{3}-1.77\cdot\left[ \frac{x_{\text{age }}-35}{14.11} \right]_{+}^{3} \\ x_{MUAC'} & =\left[ \frac{x_{\mathrm{MUAC}}-185}{20.82} \right]_{+}^{3}+1.375\cdot\left[ \frac{x_{\mathrm{MUAC}}-280}{20.82} \right]_{+}^{3}-2.375\cdot\left[ \frac{x_{\mathrm{MUAC}}-240}{20.82} \right]_{+}^{3} \\ x_{SysBP'} & =\left[ \frac{x_{\mathrm{SysBP}}-70}{13.572} \right]_{+}^{3}+0.667\cdot\left[ \frac{x_{\mathrm{SysBP}}-120}{13.572} \right]_{+}^{3}-1.667\cdot\left[ \frac{x_{\mathrm{SysBP}}-90}{13.572} \right]_{+}^{3} \\ \text{ where }[x]_{+} & =\left\{ \begin{matrix} x & \text{ if }x>0 \\ 0 & \text{ otherwise } \end{matrix} \right. \end{matrix}$$
